# The influence of biophysical parameters in a biomechanical model of cortical folding patterns

**DOI:** 10.1038/s41598-021-87124-y

**Published:** 2021-04-08

**Authors:** Xiaoyu Wang, Julien Lefèvre, Amine Bohi, Mariam Al Harrach, Mickael Dinomais, François Rousseau

**Affiliations:** 1 IMT Atlantique, LaTIM U1101 INSERM, UBL, Brest, France; 2 Institut de Neurosciences de la Timone, Aix-Marseille Université, CNRS UMR7289, Marseille, France; 3 Aix-Marseille Université, Université de Toulon, CNRS, LIS, Marseille, France; 4 Université de Rennes 1, Laboratoire Traitement du Signal et de l′Image (LTSI), INSERM U1099, F-35000 Rennes, France; 5 Université d’Angers, Laboratoire Angevin de Recherche en Ingénierie des Systèmes (LARIS) EA7315, 49000 Angers, France; 6 CHU Angers, Département de Médecine Physique, Angers, France

**Keywords:** Biological physics, Mechanical engineering, Applied mathematics

## Abstract

Abnormal cortical folding patterns, such as lissencephaly, pachygyria and polymicrogyria malformations, may be related to neurodevelopmental disorders. In this context, computational modeling is a powerful tool to provide a better understanding of the early brain folding process. Recent studies based on biomechanical modeling have shown that mechanical forces play a crucial role in the formation of cortical convolutions. However, the effect of biophysical parameters in these models remain unclear. In this paper, we investigate the effect of the cortical growth, the initial geometry and the initial cortical thickness on folding patterns. In addition, we not only use several descriptors of the folds such as the dimensionless mean curvature, the surface-based three-dimensional gyrification index and the sulcal depth, but also propose a new metric to quantify the folds orientation. The results demonstrate that the cortical growth mode does almost not affect the complexity degree of surface morphology; the variation in the initial geometry changes the folds orientation and depth, and in particular, the slenderer the shape is, the more folds along its longest axis could be seen and the deeper the sulci become. Moreover, the thinner the initial cortical thickness is, the higher the spatial frequency of the folds is, but the shallower the sulci become, which is in agreement with the previously reported effects of cortical thickness.

## Introduction

Human brain growth is accompanied by the folding of the cerebral cortex, which takes place in a hierarchical mode during gestational weeks 16–40^1^, with primary folds forming the earliest and highly conserved, then secondary folds elaborating on these folds, etc^2,3^. Recent studies have revealed that not only the molecular and cellular processes but also mechanical forces play an important role in the formation of the gyral and sulcal convolutions^4–7^.

It has been revealed that mechanical models based on the hypothesis of differential tangential growth could produce realistic folding patterns when they are applied on human fetal brain data^6,7^. 3D numerical simulations of brain growth demonstrate that the relative tangential expansion of the cerebral cortex constrained by the white matter generates compressive stress, resulting in cusped sulci and smooth gyri similar to those in developing fetal brains^6,7^.

It has been shown that the cortical folding patterns are influenced by various physical parameters, e.g., the initial cortical thickness^5–9^, the initial geometry^10–12^ and the relative growth^2,5,13–16^. In addition to these recent observations, many questions are still open regarding the morphogenesis of folding patterns, including links between the physical parameters of simulation models and the folding patterns observed in in vivo MRI data. A deeper understanding of these parameters can significantly contribute to comprehend pathologies associated with characteristic changes in cortical folding. For instance, polymicrogyria, pachygyria and lissencephaly malformations can be accompanied by autism^17,18^, schizophrenia^19,20^ or epilepsy^21^. Thus, in this work, we investigate the influence of physical parameters on surface morphology using the brain growth model proposed by Tallinen et al.^6,7^. The simulation results first allow us to visually remark the difference in the appearance of folding patterns. Then we quantify these folds through various quantitative metrics, such as the mean curvatures, the surface-based three-dimensional gyrification index and the sulcal depth, which can be used to describe the complexity degree of surface morphology^22,23^. Besides we introduce a novel approach to measure the anisotropy of the folding orientation, through geometric tools^24,25^ and the Kullback-Leibler divergence.

Specifically, this work attempts to answer the following questions: (1) What is the impact of the temporal cortical growth model onto the folding patterns? (2) What is the influence of the initial cortical thickness on the folding patterns of the brain? (3) Is there a relationship between the folding complexity (as measured by the average of the absolute value of mean curvatures, the surface-based three-dimensional gyrification index and the sulcal depth) and the shape of the brain (the initial geometry)? (4) Does the orientation of the folds depend on the shape of the brain?

## Methods

### Biomechanical model of brain folding

Tallinen et al. proposed a human cortical folding model, which can mimic a realistic brain folding process^6,7^. Brain growth is modeled by a relative tangential expansion of the cortical layer and the white matter layer, the cortical layer is assumed to grow more rapidly than the white matter layer. The nonlinear stress-strain property of the human brain^26^ and the bulk modulus (K) that is assumed to be five times the shear modulus^7^ can be brought into the frame of a modestly compressible Neo-Hookean material solid. The model is based on an explicit dynamic solver for quasi-static equilibrium of the system and allows the simulation of the large strains and highly nonlinear mechanics involved in gyrification, but the brain solid should be discretized into high-density tetrahedral finite elements^6^. In our simulation, we make use of dense meshes ($10^{6}$ tetrahedra/cm$^{3}$) to ensure the folding accuracy, which is detailed in additional information. In addition, the time step $dt = 0.05a\sqrt{\rho /K}$ is set to avoid computational instabilities^27,28^, where *a* is mesh spacing which should be set manually based on the average spacing in the mesh, $\rho$ is mass density and *K* is bulk modulus.

The model uses free boundary conditions and the initial displacement and velocity are zero. Two main forces are considered in this model. One is the elastic force, which is derived from the volumetric strain energy density of neo-Hookean and a deformation gradient. Another is the contact force, which takes place when a separation between a node and a triangle face at the brain surface is less than a threshold in order to prevent nodes from penetrating element faces. The contact force is obtained via penalty based vertex-triangle contact processing^29^.

The deformation gradient is defined in this model by $F = A(G{\hat{A}})^{-1}$, which differs from the traditional definition of $F = A{\hat{A}}^{-1}$ by integrating the relative tangential growth tensor *G*. The relative tangential growth tensor *G*, which describes the tangential expansion perpendicular to the normal vector ${\hat{n}}$ of the tetrahedron, is calculated by

1 $$\begin{aligned} G = gI+(1-g){\hat{n}}\otimes {{\hat{n}}}, \end{aligned}$$

where *g* is the relative tangential expansion ratio of the grey matter to the white matter, which associates with the distance of a tetrahedron from surface in material coordinates and is given by the relation

2 $$\begin{aligned} g = 1 + \frac{\alpha _{t}}{1+e^{10(\frac{y}{H_{i}}-1)}}, \end{aligned}$$

where $\alpha _t$ controls the magnitude of local cortical expansion (expansion for each tetrahedron). *t* parametrizes time of model and has a non-linear relation to gestational age (*GA*) as $t = 6.926\times 10^{-5}GA^3-0.00665GA^2+0.250GA-3.0189$^9^, $t\in [0,1]$ corresponds to $GA\in [22 weeks, adult]$, *y* is the distance from the top surface, which is calculated for four vertices of each tetrahedron and would be averaged, $H_i$ is the initial cortical thickness. When the relative tangential growth tensor *G* is initialized, the brain solid starts to grow and the deformation gradient is formed, then the corresponding elastic force can be calculated. The resultant force (the sum of the elastic force and the contact force) is applied as the nodal force on each node of the mesh to produce the deformation of the brain solid. The Python code used in this study is available at https://github.com/rousseau/BrainGrowth.

### Biophysical and numerical parameters

To explore the mechanical mechanisms leading to folding, simulations are performed on an ellipsoid with mesh density of $10^{6}$ tetrahedra/cm$^{3}$. The biophysical parameters defined in the model, such as the initial geometry, the initial cortical thickness ($H_i$) and the cortical growth ($\alpha _t$), may affect the folding patterns on soft solids. For the definition of the initial cortical thickness ($H_i$), we should consider the size of the initial geometry. The two equatorial radius and polar radius of the ellipsoid are approximately 10, 9 and 7 mm respectively, thus the longitudinal length (LL) of this ellipsoid is 20 mm. For a 22 weeks’ normal fetal brain, the brain longitudinal length (BLL) is approximately 60 mm^30–32^, and the typical cortical thickness is 2.5 mm^7^. To respect the ratio of the initial cortical thickness to the longitudinal length, a scale factor is applied to obtain the initial cortical thickness of the ellipsoid, which is 0.83 mm. It should be noted that the model has a coordinates normalization part (the three-dimensional coordinates will be $\in [-1, 1]$), thus the initial volume of the solid does not affect simulation results, and the initial cortical thickness will be normalized to 0.042.

For the cortical growth ($\alpha _t$ in Eq. 2), at $t=1$ (adult brain) the cortical layer has an areal growth by a factor of $g^{2} = 8$ relative to the white matter zone in the victitious stress-free state^7^, thus the linear cortical growth was originally defined as $\alpha _{t} = (\sqrt{8}-1)t = 1.829t$ in this model.

#### Cortical growth

**Figure 1:**
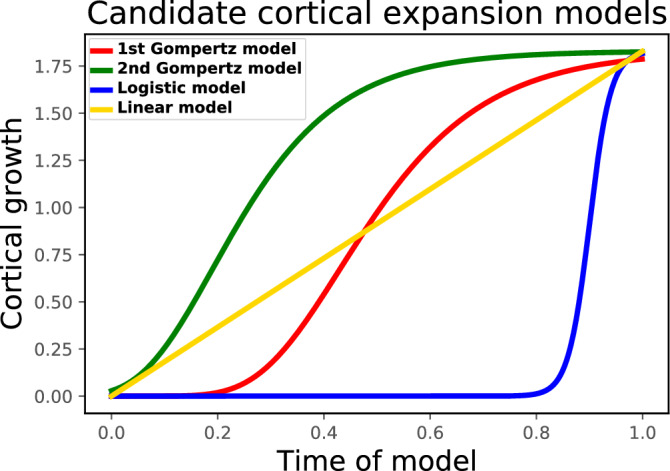
Curves of the cortical growth defined by different expansion models.

The cortical growth (defined by $\alpha _t$ in the model) has an effect on surface morphology^2,5,13–16^. Different growth models, which are used to describe different change tendencies of the cortical growth, may also have an impact onto the folding patterns. To better understand it, we first use a linear growth model, which was initially defined in the brain folding model by $\alpha _t = (\sqrt{8}-1)t$^7^. Secondly, considering that the Gompertz distribution can be used to model the growth of human brains^33^, we choose the Gompertz growth model to compare with the linear growth model, which is defined as:

3 $$\begin{aligned} \alpha _t = ae^{-e^{-b(t-c)}}, \end{aligned}$$

where *a* is the asymptotic value, *b* sets the growth rate and *c* sets the displacement along the t-axis. Since the purpose is to explore the effect of change tendency of the growth, the initial and final values of the Gompertz growth model should be in agreement with those of the linear growth model, thus we assume *a* is a constant parameter ($\sqrt{8}-1$). With (*b*, *c*) equal to (6.6, 0.43) and (7.5, 0.19), we define two growing modes which correspond to the 1st and 2nd Gompertz models shown in Fig. 1. In addition, another aim is to know how the extreme growth mode of non-growth for a long time and rapid growth at the end of the simulation to reach the same final growth will affect the folding patterns, thus the logistic model is adopted:

4 $$\begin{aligned} \alpha _t = \frac{a}{1+e^{-b(t-c)}}, \end{aligned}$$

where *a* is curve’s maximum value, *b* is the logistic growth rate and *c* is the *t* value of the sigmoid’s midpoint. With $a=\sqrt{8}-1$, $b=50$ and $c=0.9$, the model allows to grow rapidly around time 0.9. Eventually, these growth models are integrated into the brain folding model respectively to perform the simulations on the ellipsoid.

#### Initial geometry and cortical thickness

The pattern and location of folds can be influenced by initial geometry^10–12^. For example, in ellipsoid models, most folds run either parallel or orthogonal to the ellipsoid’s long axis^10,11^. In order to understand more clearly, an affine transformation (elongated transformation) is applied to the initial geometry to determine whether the complexity of folding patterns and how the direction of folds will change. Therefore, we propose that, while keeping the volume and the y-axis length of the geometry unchanged, the reference ellipsoid is scaled in x and z directions to obtain a sphere and the ellipsoids with different elongation ratios. The elongation ratio is defined by the ratio of the x-axis length to z-axis length. The y-axis length of these geometries is 18 mm, the x-axis length is from 19 to 27 mm, the z-axis length is from 18 to 12 mm, and the corresponding elongation ratio varies from 1.0 to 2.25.

In addition, the cortical folding patterns can also be influenced by the initial cortical thickness^5–7,9^. To understand the effect of the initial cortical thickness on surface morphology, based on each geometry with the linear growth model $\alpha _t = 1.829t$, we vary the initial cortical thickness in the brain folding model from 0.03 to 1.63 mm (0.03, 0.43, 0.63, 0.83, 1.03, 1.23 and 1.63 mm) to simulate the folding processes. The cortical thicknesses from 0.43 to 1.23 mm are defined according to normative human cerebral cortex measurements^34^ and the scale factor of the longitudinal length which is introduced in the Section of biophysical and numerical parameters. The other two cortical thicknesses (0.03 and 1.63 mm) are the hypotheses for abnormal cortical thicknesses.

### Quantitative methods

#### Curvatures on triangle meshes

The normal curvature on a 3D surface in some direction is the inverse of the radius of the circle that best approximates a surface normal slice in that direction^35^. The normal curvature for a smooth surface can be represented by the Weingarten matrix, i.e. the second fundamental tensor **II**, which is defined in terms of the directional derivatives of the surface normal:

5 $$\begin{aligned} \mathbf{II}= \begin{pmatrix} D_{u}n&D_{v}n \end{pmatrix} = \begin{pmatrix} \frac{\partial n}{\partial u} \cdot u &{} \frac{\partial n}{\partial v} \cdot u \\ \frac{\partial n}{\partial u} \cdot v &{} \frac{\partial n}{\partial v} \cdot v \end{pmatrix}, \end{aligned}$$

where (*u*, *v*) are the directions of an orthonormal coordinate system in the tangent frame (the sign convention used here produces positive curvatures for convex surfaces with outward-facing normals).

In this study, we compute the curvature based on Rusinkiewicz estimation^22^, which may be thought of as an extension of common methods, such as the curvature presented in Knutsen et al.^36^ and used by subsequent authors like Garcia et al. ^37^, for the purpose of estimating per-vertex normals by averaging adjacent per-face normals. This algorithm uses the “Voronoi area” weighting which can produce more accurate normal estimates of curvature than other weighting methods for triangles of varying sizes and shapes. In this algorithm, the per-face (per-triangle) curvature tensor is first computed by its three well-defined directions (the edges) together with the differences in normals in those directions (computed from the per-vertex normals). Then, the algorithm performs a coordinate system transformation for converting the curvature tensor to the vertex coordinate frame. Eventually, a per-face coefficient is applied to allow to weight the face curvature around each vertex.

Mean curvature of a vertex is defined by the average of the two principal curvatures (the maximal and minimal curvatures) of the vertex, and the principal curvatures are the eigenvalues of the vertex normal curvature tensor computed by Rusinkiewicz estimation:

6 $$\begin{aligned} \mathbf{II} = \begin{pmatrix} u'&v' \end{pmatrix} \begin{pmatrix} K_1 &{} 0 \\ 0 &{} K_2 \end{pmatrix} \begin{pmatrix} u' \\ v' \end{pmatrix}, \end{aligned}$$

where $K_1$ and $K_2$ are the eigenvalues and $(u',v')$ are the principal directions, which are the directions in which the normal curvature reaches its minimum and maximum. Since surface curvature is useful to describe spatial variations in folding, thus for the overall folding complexity comparison, we first compute a dimensionless mean curvature by multiplying the mean curvature by the square root of the surface area ($K\sqrt{area}$), where *K* is the mean curvature. Then we calculate the average (across all vertices on the mesh surface) of the absolute value of dimensionless mean curvatures (at a vertex on the mesh surface) for each simulated surface. In the remainder of the manuscript, we simply use the term curvature for the sake of clarity.

#### Three-dimensional gyrification index

Curvature-based features do not provide complete description of the folding patterns. In order to describe globally the folding complexity by considering the depth and wideness of the cortical folding, we also use the surface-based three-dimensional gyrification index (3D GI). It is a global measurement which is defined as the ratio of the cortical surface area to the area of its smooth “convex hull” (the minimum surface area needed to completely enclose the brain)^23^:

7 $$\begin{aligned} 3D\;GI = \frac{{area of cortical surface}}{{area of convex hull}}. \end{aligned}$$

To get the convex hull, we scale the initial smooth surface in three dimensions so that the three-dimensional lengths of the convex hull are equal to those of the simulated cortical surface.

#### Sulcal depth

Sulcal depth can be used as a quantitative marker of cortical morphology^38^. Several approaches have been proposed to compute the sulcal depth^39–41^ but a well-defined computation of depth remains an open question. In this work, we make use of an intuitive approach to calculate the sulcal depth by using the distance between the deformed mesh surface and the corresponding convex hull. Specifically, for each surface vertex of the deformed mesh, we find the intersection point on the convex hull by using the vector determined by the corresponding vertex of the initial mesh and this vertex and the method of traversing all triangles on the convex hull. Then we compute the distance between each surface vertex of the deformed mesh and its corresponding intersection point on the convex hull.

#### Folds orientation

For the purpose of describing and comparing the direction of the folds on the simulated surfaces, we calculate the angle between the gradient of Fiedler vectors^24^ and the principal directions of curvatures^25^, which helps to understand whether the folds are isotropic. The Fiedler vector is the first non-constant eigenfunction of Laplace-Beltrami operator, represented by $\phi _{1}$ in Eq. 8^24^. The Laplace-Beltrami operator is defined as $\Delta _{M} = div\cdot \nabla _{M}$, where *M* is a Riemannian manifold. The eigenvalues of $-\Delta _{M}$ are $\lambda _{0} = 0\le \lambda _{1}\le \ldots$ and $\phi _{0}$, $\phi _{1}$, $\ldots$ are associated orthonormal basis of eigenfunctions, which satisfy

8 $$\begin{aligned} -\Delta _{M}\phi _{i} = \lambda _{i}\phi _{i}. \end{aligned}$$

The Fiedler vector allows to describe the longitudinal extension of surfaces^24,42–44^. The Fiedler’s extrema are the most distant points^24^, and its contour lines are slices in the elongation axis. The gradient of the Fiedler vector that is perpendicular to the contour lines gives the direction of elongation. The principal directions of curvatures are the corresponding eigenvectors of the principal curvatures (the eigenvalues of the Weingarten matrix). Based on the local scalar product between the gradient of the Fiedler vector and the principal directions of curvatures, we can obtain the folds angle.

In order to compare quantitatively the uniformity of the angular distribution of folds, we use the Kullback–Leibler (KL) divergence. The KL divergence, also called relative entropy, is used to measure how one probability distribution is different from a second reference probability distribution. For two discrete probability distributions P and Q defined on the same probability space, the KL divergence from P to Q is defined to be

9 $$\begin{aligned} D_{KL}(P || Q) = \sum _{i} P(i)\log \frac{P(i)}{Q(i)}. \end{aligned}$$

For angular uniformity calculations, P corresponds to the fold angular distribution on the folded surface, Q represents the theoretically uniform distribution of fold angles.

## Results

### Cortical growth

#### Surface morphology and 3D GI

**Figure 2:**
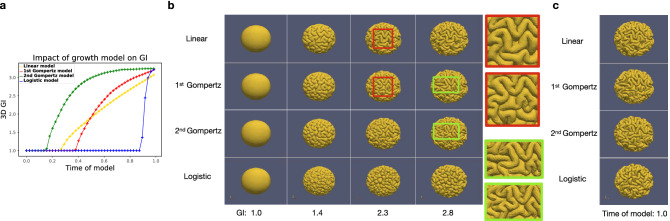
(**a**) The evolution of 3D GI over time for surfaces of different expansion models. (**b**) The comparison of folding patterns on the reference ellipsoid at the same 3D GI based on different expansion models. In red frames: difference in folding patterns for linear and 1st Gompertz models; in green frames: difference in folding patterns for 1st and 2nd Gompertz models. (**c**) The comparison of folding patterns on the reference ellipsoid at time 1.0 based on different expansion models. Software: (**b**, **c**) ParaView-5.8.0, https://www.paraview.org/.

Based on the simulation results of the reference ellipsoid with the same initial cortical thickness 0.83 mm but different growth models, we first compute the 3D GI over time, as shown in Fig. 2a. The increasing tendencies of the 3D GI for the different growth models are consistent with the curves of the cortical growth shown in Fig. 1. The final 3D GI is almost the same for the models, showing that different growth models with the same initial and final growth will almost not affect the complexity of the final surface morphology.

For the 3D GI of 1.0, 1.4, 2.3 and 2.8, the surface morphology of different growth models is shown in 2b. When the 3D GI is 1.4, the folds on the surfaces of different growth models are almost in the same position. However, the divergence of folding patterns takes place when the 3D GI reaches 2.3 for the linear and 1st Gompertz model (in red frames) and is 2.8 for the 1st and 2nd Gompertz model (in green frames), suggesting that the same type of expansion model can make the difference in folds occur relatively late. For the logistic expansion model, the folds are relatively shorter compared to those of other models since the cortical growth is zero for a long time and suddenly increases close to the end. The final results (at time 1.0) of different growth models are shown in Fig. 2c. The amount of the final folds is almost the same for these growth models, but the patterns of the folds are visually different.

#### Surface curvature

**Figure 3:**
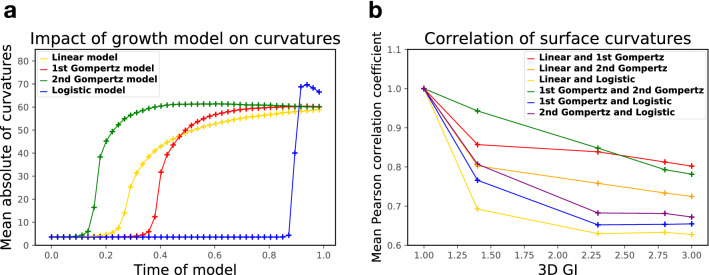
The comparison of (**a**), average of absolute value of dimensionless mean curvatures and (**b**), mean Pearson correlation coefficients of mean curvatures for surfaces based on different expansion models.

The impact of growth model on dimensionless curvature is reported in Fig. 3a. For the 2nd Gompertz growth model, the curvature increases and reaches its maximum earlier than that of other models. For the logistic model, the curvature suddenly increases towards the end of the simulation. The final value of the curvature (at time of model 1.0) is almost the same for these growth models.

Furthermore, in order to quantify the correlation of the folding patterns generated by different growth models under the same surface folding complexity, at the same 3D GI, we calculate the Pearson correlation coefficient of mean curvature on each vertex of the surfaces produced by every two different expansion models. The average of the correlation coefficients on all vertices is shown in Fig. 3b. The average correlation coefficients of surface curvatures between different growth models are all higher than 0.6. Especially for the linear and 1st Gompertz models, and 1st and 2nd Gompertz models, the average correlation coefficients are as high as 0.8 after all folds are formed. It can also be seen that before the 3D GI reaches 2.45, the average correlation coefficient of the surfaces generated by 1st and 2nd Gompertz growth models is higher than that of the others, which is consistent with the similar folding patterns of the two Gompertz models shown in Fig. 2b.

### Initial geometry and cortical thickness

#### Surface morphology

**Figure 4:**
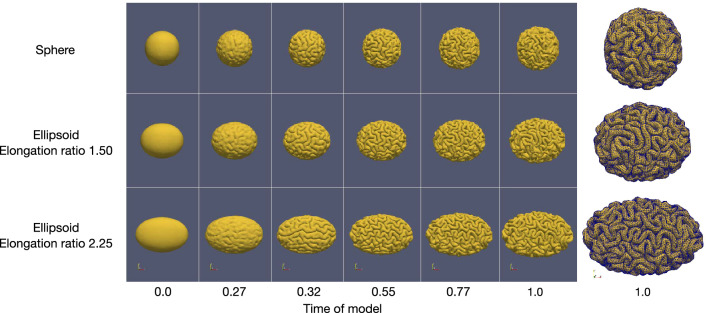
The comparison of folding patterns on different geometries with the same initial cortical thickness. Software: ParaView-5.8.0, https://www.paraview.org/.

The simulation results for the geometries of the elongation ratios 1.0, 1.50 and 2.25 with the initial cortical thickness 0.83 mm are shown in Fig. 4. We can observe that, at time 0.27, the primary folds on geometries of different elongation ratios appear almost at the same positions, but the orientation of the folds begins to differ over time. After time 0.55 when most of the folds have already been formed, the size and spatial frequency of the folds are almost the same for the three geometries.

**Figure 5:**
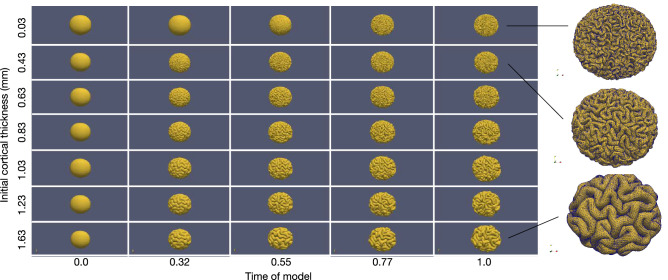
The comparison of folding patterns on reference ellipsoid for different initial cortical thicknesses. Software: ParaView-5.8.0, https://www.paraview.org/.

The simulated surfaces of the reference ellipsoid with the initial cortical thickness varying from 0.03 to 1.63 mm are shown in Fig. 5. The surface with the thinnest initial cortical thickness 0.03 mm folds relatively late than the others. In addition to the surface of the thinnest initial cortical thickness, at time 0.32, other surfaces already showed some clear primary folds, and the thicker the cortex, the more obvious the folds are; starting from time 0.55, most of gyri and sulci are formed for all of these thicknesses. As the initial cortical thickness increases, the gyri become larger and the folds become fewer, thus the adjacent sulci are more isolated; after time 0.77, the folds are almost no longer complex. The final surface morphology of the thinnest cortical thickness (0.03 mm) resembles the folding patterns of polymicrogyria^5,45^, while the surface morphology of the thickest cortical thickness (1.63 mm) is similar to the phenomenon of pachygyria.

#### Quantitative effect on folding complexity

**Figure 6:**
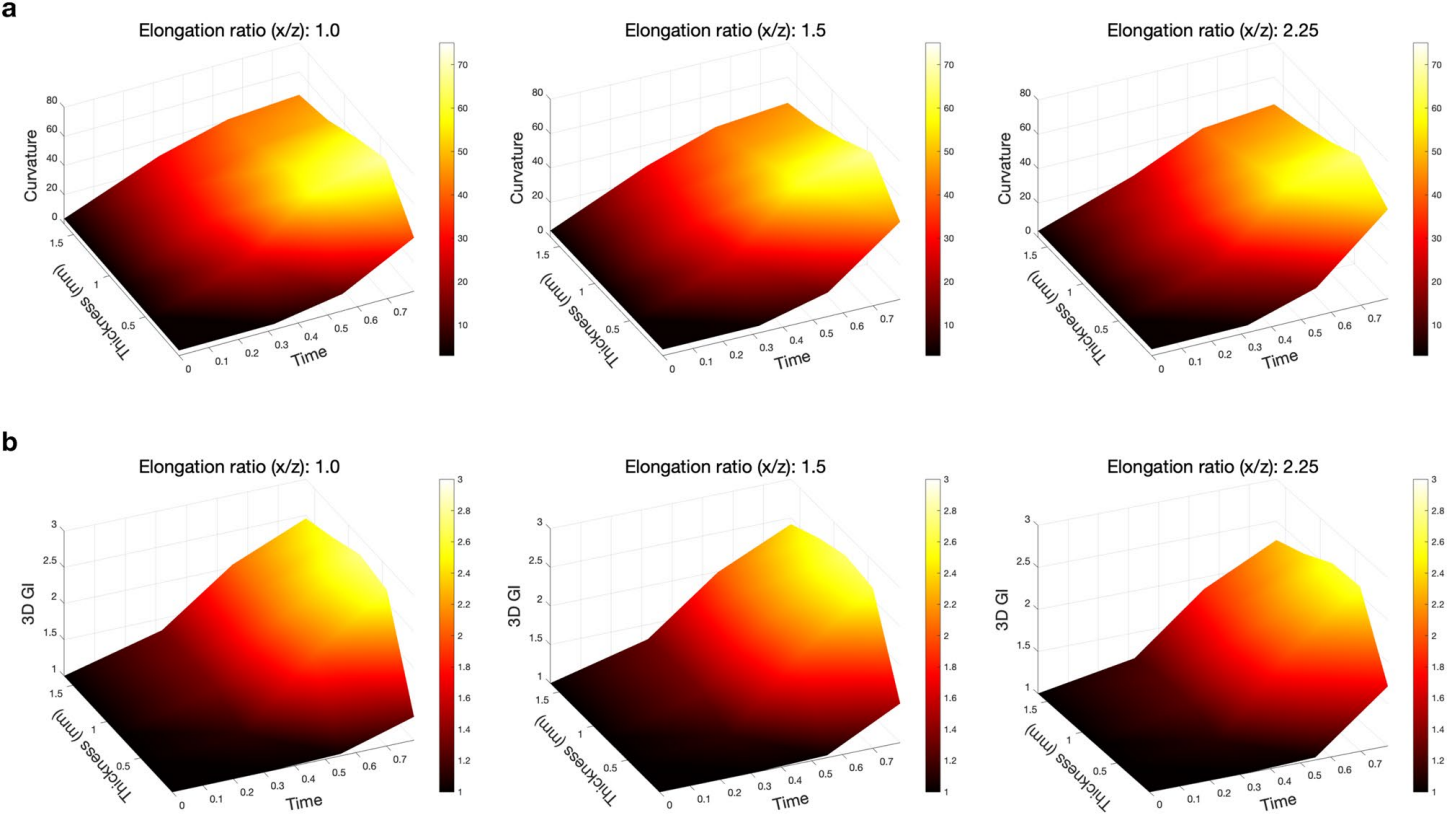
The comparison of (**a**), average of absolute value of dimensionless mean curvatures and (**b**), 3D GI over time for geometries with different initial cortical thicknesses and elongation ratios.

The folding complexity is quantitatively evaluated using the dimensionless curvature, 3D GI and the sulcal depth. Figure 6a shows the dimensionless curvature results for different cortical thicknesses and elongation ratios. It is found that the mean curvatures almost not depend on the elongation ratio (i.e., the initial geometry), but depend only on the initial cortical thickness. For the initial cortical thicknesses between 0.43 and 1.63 mm, the thinner the initial cortical thickness is, the more quickly the curvature increases after time 0.32, and the greater the curvature becomes. For the thinnest initial cortical thickness 0.03 mm, the curvature is smaller than that of the others before time 0.55; Starting from time 0.55, it increases faster, and eventually becomes almost the same or even greater than the curvature corresponding to the thicker initial cortical thicknesses.

The comparison of the 3D GI calculated on the surfaces of different cortical thicknesses and elongation ratios is shown in Fig. 6b. Likewise, the 3D GI does not depend on the elongation ratio, but on the initial cortical thickness. For the initial cortical thicknesses between 0.43 and 1.63 mm, the increment of 3D GI becomes smaller as the initial cortical thickness increases. When the initial cortical thickness is overly thin (0.03 mm), the increment of 3D GI is less than that of the other cortical thicknesses. These quantitative measurements demonstrate that when the initial cortical thickness is within a reasonable range, the thinner the initial cortical thickness is, the more complex the folding patterns become, which confirms the previously reported effects of cortical thickness^5,9^.

**Figure 7:**
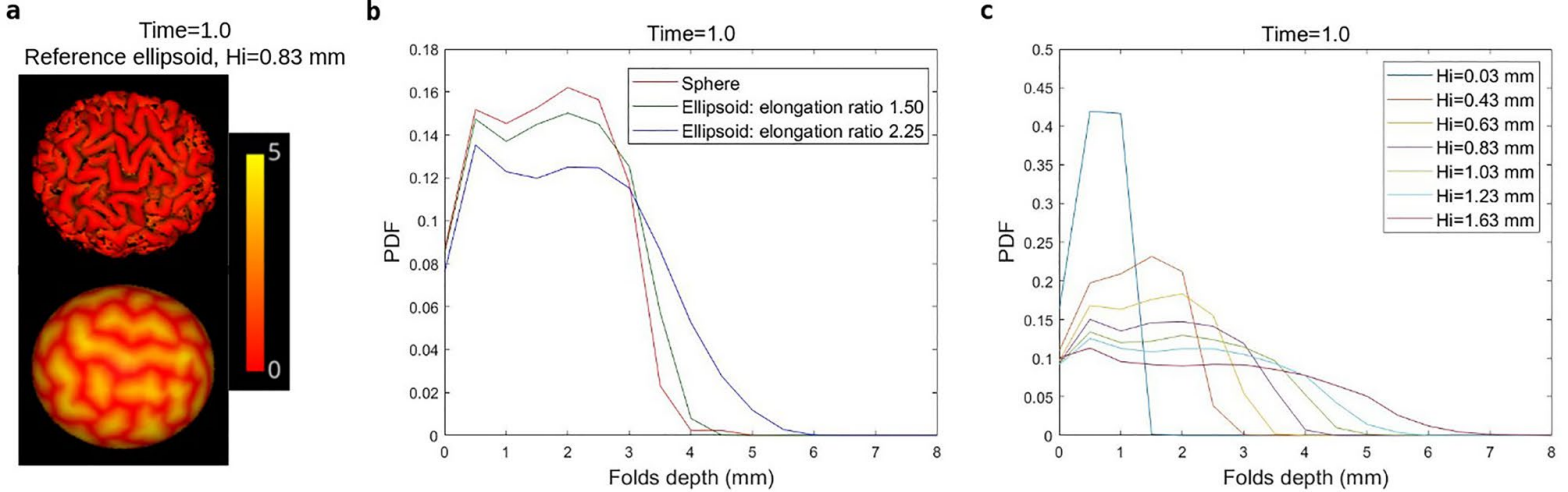
The effect of initial geometry and initial cortical thickness ($H_{i}$) on sulcal depth. (**a**), visualization of sulcal depth at time 1.0 on the deformed and corresponding initial reference ellipsoid with the initial cortical thickness 0.83 mm, (**b**), probability density function (PDF) of sulcal depth at time 1.0 for different initial geometries, (**c**), PDF of sulcal depth at time 1.0 for different initial cortical thicknesses. Software: (**a**), Visbrain-0.4.5, http://visbrain.org/.

The folding process can also be quantified by studying the sulcal depth. Figure 7 shows, as an example, a visual depth map of the folded reference ellipsoid and histograms of depth with respect to different initial geometries and cortical thicknesses. It can be clearly seen that the geometry has an effect on sulcal depth. The simulated sulci on the ellipsoids are deeper than in the case of the sphere, the greater the elongation ratio is, the deeper the sulci become. Combining the results in Figs. 4, 5 and 6, we can conclude that the elongation ratio of the initial geometry does almost not change the surface curvature and 3D GI, but it has an impact on sulcal depth. Moreover, the thinner the cortex is, the shallower the sulci become, which is in agreement with the analysis of sulcation morphology in polymicrogyria^46^.

#### Effect on folding orientation

**Figure 8:**
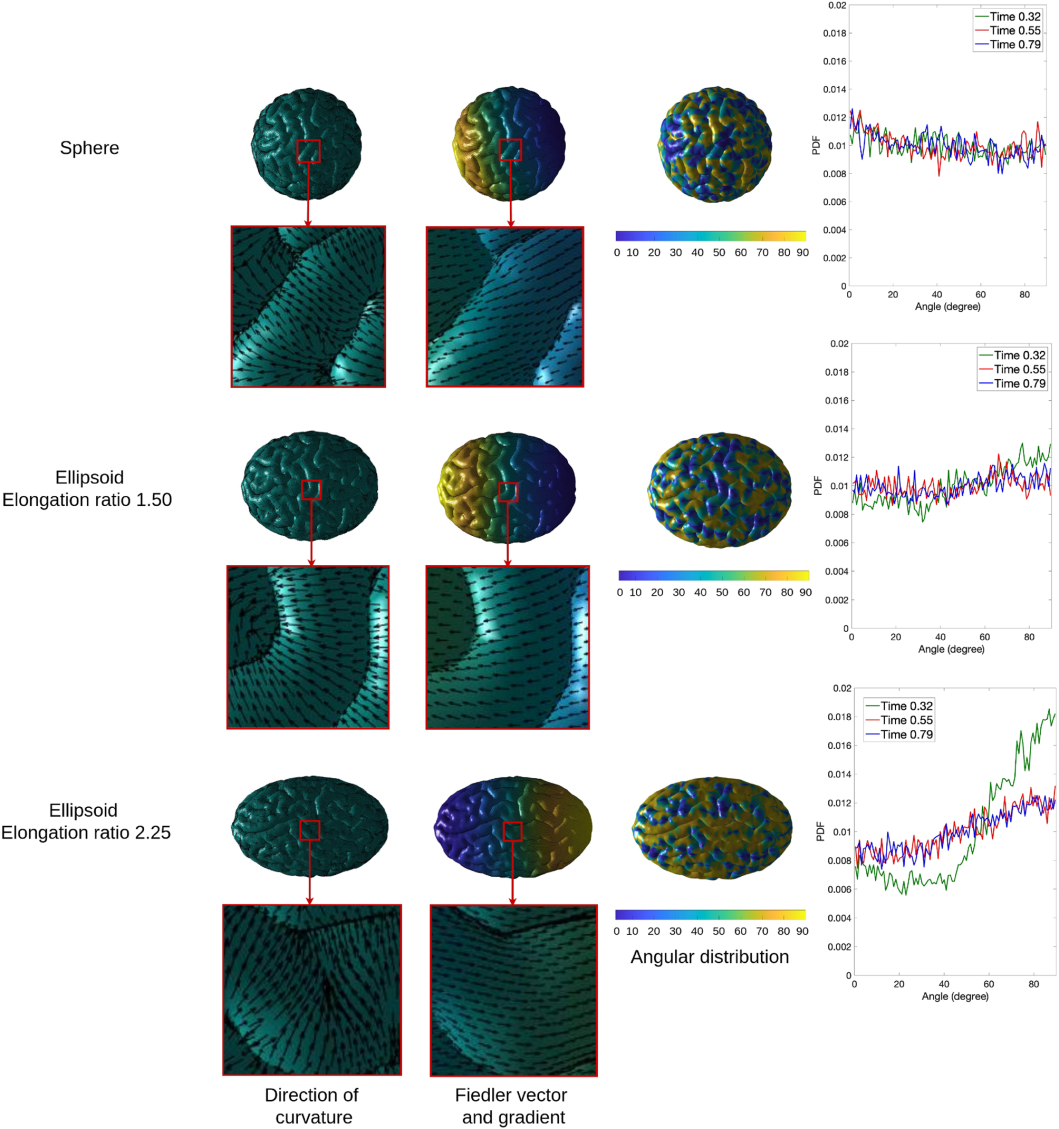
Illustration of principal directions of curvatures, gradients of Fiedler vectors, and angular distributions for surface of sphere, ellipsoid of elongation ratio 1.50 and ellipsoid of elongation ratio 2.25 at time 0.32, 0.55 and 0.79. Software: MATLAB R2019b, https://www.mathworks.com/.

To understand the effect of the initial geometry on the folds orientation, we calculate the angle between the gradient of Fiedler vectors and the principal direction of curvatures on the surface of each geometry. The angular distributions for the geometry of the elongation ratio 1.0, 1.50 and 2.25 with the same initial cortical thickness 0.83 mm at time 0.32, 0.55 and 0.79 are shown in Fig. 8. It is clear that at time 0.32, the distribution of the fold angles is almost uniform on the sphere and slightly nonuniform on the ellipsoid of the elongation ratio 1.50, while following a privileged direction on the ellipsoid of the elongation ratio 2.25. Since the direction of curvatures is perpendicular to the extension of folds, and the gradient of Fiedler Vector is along the longitudinal extension of surfaces, the peak appearing at around $90^{\circ }$ indicates the most of folds are along the longitudinal axis of the ellipsoid of the elongation ratio 2.25, which is consistent with previous observations of the location of folds in ellipsoid models^10,11^. As time passes, the number of folds increases and the angular distribution becomes more and more uniform, especially for the ellipsoid of the elongation ratio 2.25, but it is still not as uniform as that of the sphere and the ellipsoid of the elongation ratio 1.50.

**Figure 9:**
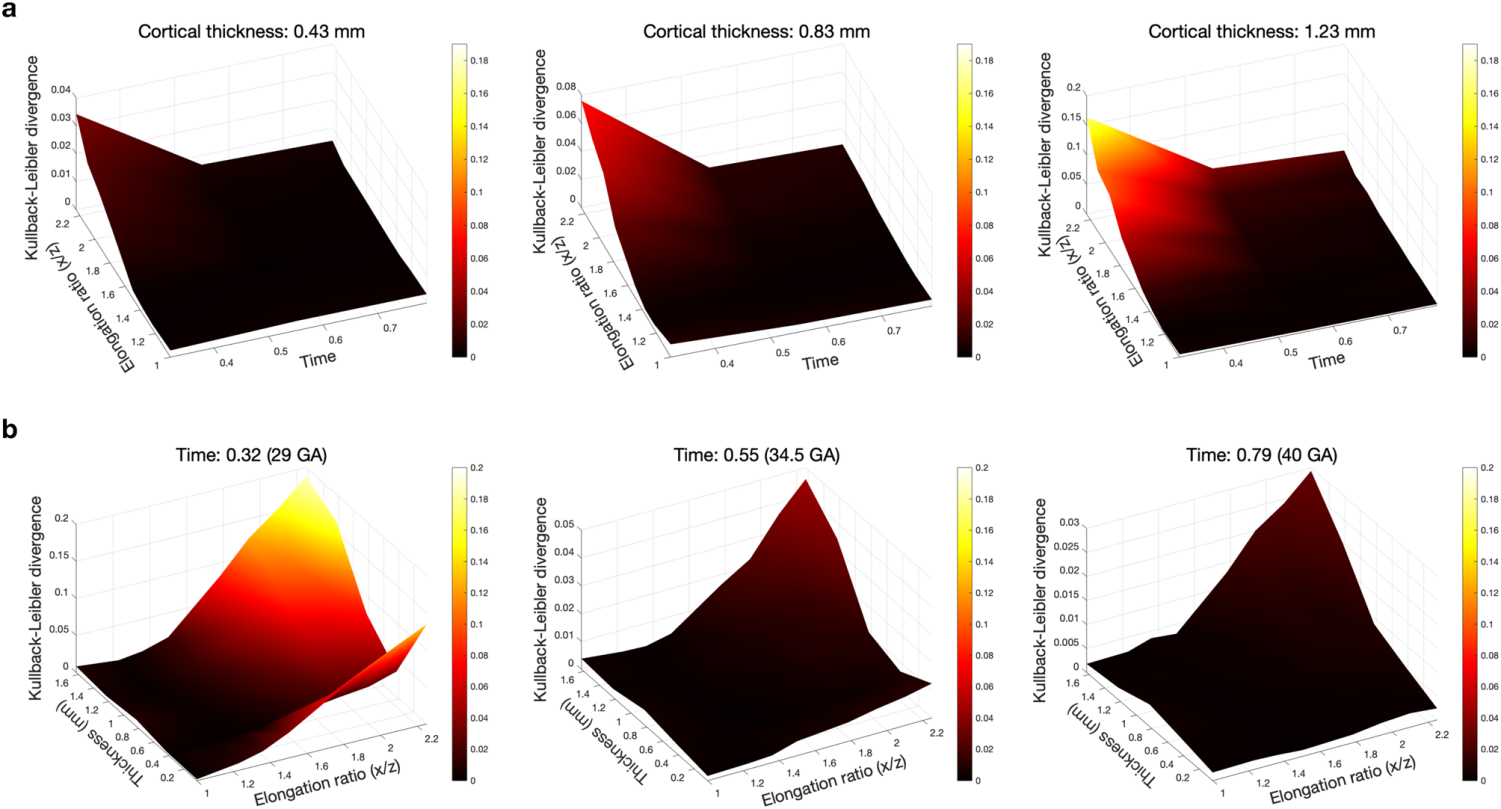
Kullback–Leibler divergence of uniformity of angular distribution for geometries with different elongation ratios and initial cortical thicknesses. Two scales are used here: the z-axis varies in each frame to allow a clear visualization of the behavior of KL divergence for a fixed initial cortical thickness (**a**)/time of model (**b**); the colorbar is the same across a row to show the evolution of KL divergence relative to uniform distribution.

To compare quantitatively the angular uniformity degree of the folding patterns for the surfaces of different elongation ratios and cortical thicknesses, the KL divergence is computed on each surface, and the results are shown in Fig. 9. The angular distribution of the folds strongly depends on the elongation ratio, and it becomes nonuniform as the elongation ratio increases, the thicker the cortex is, the more obvious this tendency becomes. As time goes on, the fold angles become more uniform especially for the geometries with larger elongation ratios.

## Discussion

The biomechanical model based on the differential tangential growth hypothesis has been used for the realistic simulation of the early expansion and folding process of the human cerebral cortex^6,7^. Therefore, such biomechanical model can be used to study the relationship between biophysical parameters and severe cortical folding malformations which are thought to be associated with neurodevelopmental diseases. In this work, we have implemented the model of Tallinen et al.^6,7^ in Python which is available at https://github.com/rousseau/BrainGrowth, used here to investigate the impacts of the several cortical growth models, the initial cortical thickness and the initial geometry onto the cortical surface morphology in an attempt to answer the questions raised in the introduction.

Regarding the impact of the temporal cortical growth model onto the folding patterns, four growth modes are defined using linear, Gompertz and logistic models to simulate the folding process. The simulation results and quantitative indices (3D GI and surface curvature) demonstrate that, when all folds are formed on the surfaces, the different growth modes with the same initial and final growth will not cause noticeable changes of the complexity degree of the folding patterns. Nevertheless, the growth mode can affect the pattern of the folds using the logistic model. This may be due to the growth rate (the slope of the growth curve in Fig. 1) at certain moments being too high to fulfill the quasi-static constraint. In addition, in a quasi-static case, even if the absolute growth rate should not affect folding, the relative growth rate of the cortex to the sub-cortical regions may have an effect on folding patterns.

In recent studies, Garcia *et al.* observed significant regional differences in growth across the cortical surface of 30 preterm infants, which are consistent with the emergence of new folds^16^, but the effect of these regional differences on folding patterns has not been quantified. Therefore, choosing a proper local growth model may be a crucial step, which can contribute to the study of the regional differential growth of cortex.

To understand the influence of the initial cortical thickness on the folding patterns of the brain, the surface morphology is studied by using five normal and two abnormal cortical thicknesses varying from 0.03 to 1.63 mm. The results show that, the thinner the initial cortical thickness is, the higher the spatial frequency of the folds appears to be, but the shallower the sulci become, which is consistent with the reported effects of the cortical thickness in previous works^5,9,46^. The final surface morphology of the cortical thickness of 0.03 mm is similar to the phenomenon of polymicrogyria, while the surface morphology of the cortical thickness of 1.63 mm resembles the phenomenon of pachygyria. These observations may be crucial for exploring the causes of autism, schizophrenia and epilepsy diseases which may be related to cortical malformations^18,20,21^.

To study whether there is a relationship between the folding complexity and the shape of the brain, we change the elongation ratio of the initial geometry from 1.0 to 2.25 while keeping the volume and the y-axis length of the geometry unchanged. The quantitative results illustrate that the variation in the shape (the elongation ratio) of the geometry has an impact on the sulcal depth, but not on the surface curvature and 3D GI.

In order to investigate whether the orientation of the folds depends on the shape of the brain, we propose to calculate the fold angles between the gradient of Fiedler vectors and the principal directions of curvatures on surfaces of different elongation ratios, and then use the Kullback-Leibler divergence to measure the anisotropy of the folding orientation. We find that the shape (the elongation ratio) of the geometry can predict the orientation of the folds (at least primary folds). The slenderer the initial geometry is, the greater the number of primary folds along its longitudinal direction becomes. In addition to the elongation ratio, other geometric changes may also affect the folding patterns, such as the longitudinal fissure of the human brain. Therefore, it’s also important to study the effect of other geometric changes on folding. The calculation method of folding orientation can be used for future works to measure the orientation of the folds on surfaces with other geometric changes.

## Additional information

### Mesh density

Mesh density is the number of elements per unit volume in a volumetric mesh. In finite element analysis, mesh density is a crucial issue, which is closely associated with the accuracy of the finite element model and determines its complexity degree. In order to investigate the effects of variations in mesh density on surface morphology, based on the same surface mesh of the reference ellipsoid, we generated volumetric meshes by Netgen from approximately $10^{2}$ to $10^{6}$ tetrahedra. They have 535, 4280, 34240, 200944, 1181216, 2314240, 3897088 and 5248576 tetrahedra, respectively. The normalized volume of these volumetric meshes is 2.5 cm$^{3}$, therefore, the mesh densities of them are approximately 214, 1712, 13696, 80378, 472486, 925696, 1558835 and 2099430 tetrahedra/cm$^{3}$.

**Figure 10:**
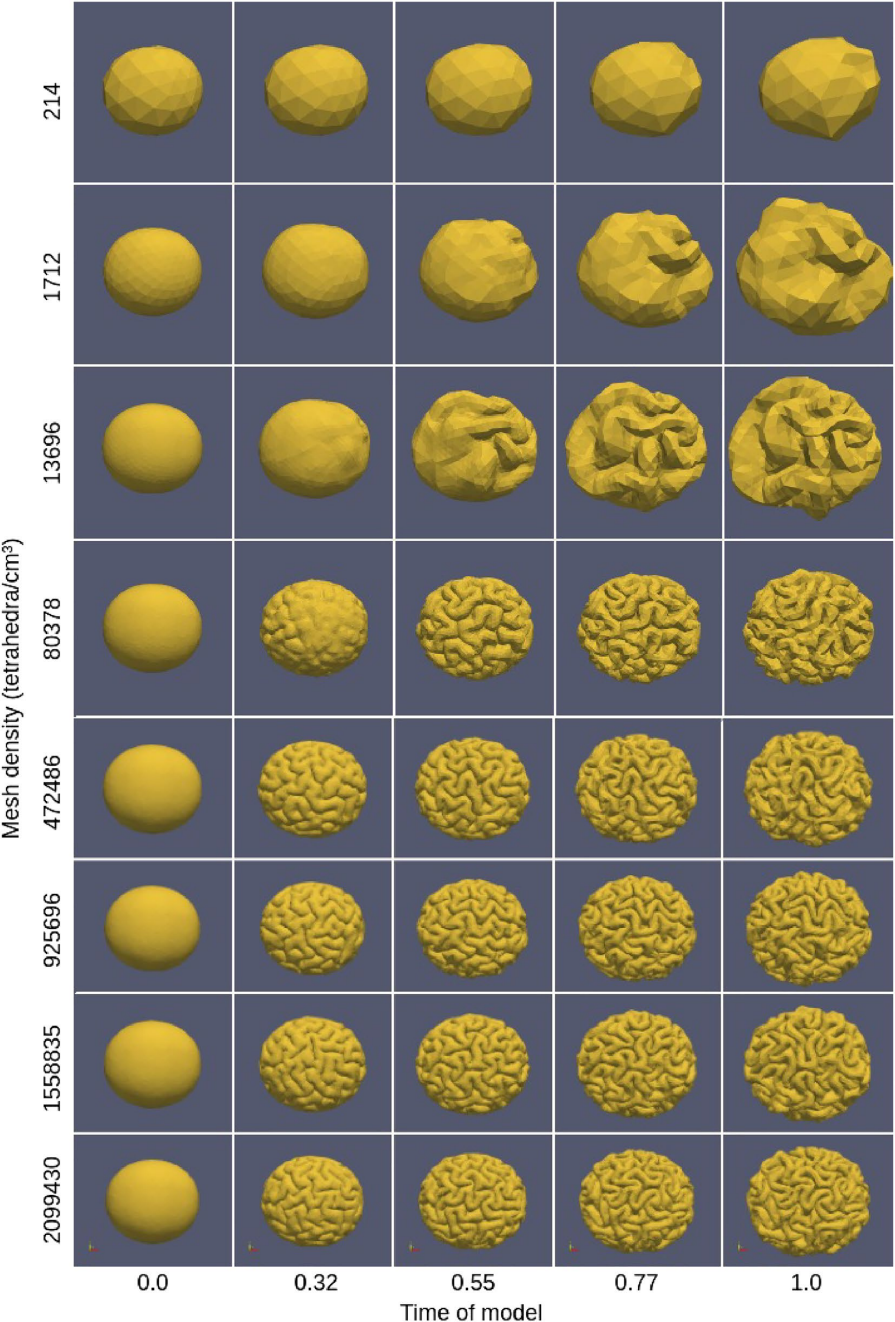
The comparison of folding processes of ellipsoids with different mesh densities. Software: ParaView-5.8.0, https://www.paraview.org/.

With the initial cortical thickness 0.83 mm and the linear growth model $\alpha _t = 1.829t$, the simulation results of the brain folding model are shown in Fig. 10. It can be observed that deformations first appeared where the sizes of elements of entire ellipsoids were the smallest, i.e., the boundary areas of ellipsoids. Moreover, the higher the mesh density is, the greater the number of folds and the smaller the width of gyri becomes. However, when the mesh density reaches approximately the order of $10^{6}$ (925696 tetrahedra/cm$^{3}$), the size and the amount of folds will rarely change anymore. That is to say, when the mesh density reaches a certain order of magnitude, further increases in mesh density will increase computational cost but cannot significantly change the spatial frequency of folding patterns. However, the folding patterns are totally different for the last three largest mesh densities, which is because the volumetric meshes of different densities that we used are different. These different perturbations in mesh can produce different patterns because they are the mechanism breaking the symmetry in the system^6^. In short, when the density of the mesh reaches a certain order of magnitude, the size of the folds tends to be stable, but the folding patterns have random spatial variations according to each mesh.

**Figure 11:**
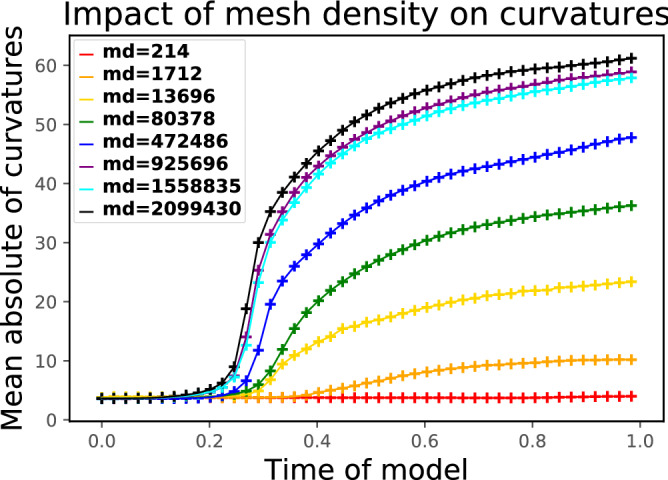
The comparison of average of absolute value of dimensionless mean curvatures for simulated surfaces of different mesh densities (md).

The comparison of the dimensionless curvatures for surfaces of different mesh densities is shown in Fig. 11. We can observe that after time 0.3, except for the mesh densities of 925696 and 1558835, the higher the mesh density is, the greater the curvature becomes. For the last three largest mesh densities, the change in the curvature is small enough (< 3.5), the error is less than 5% (3.5/61 $\approx$ 5.7%), which can be ignored. Therefore, it is possible to comprehend that when the mesh density reaches the order of $10^{6}$ tetrahedra/cm$^{3}$, the solution converges and the complexity degree of folding patterns no longer changes, the curvature oscillates around 61.

**Figure 12:**
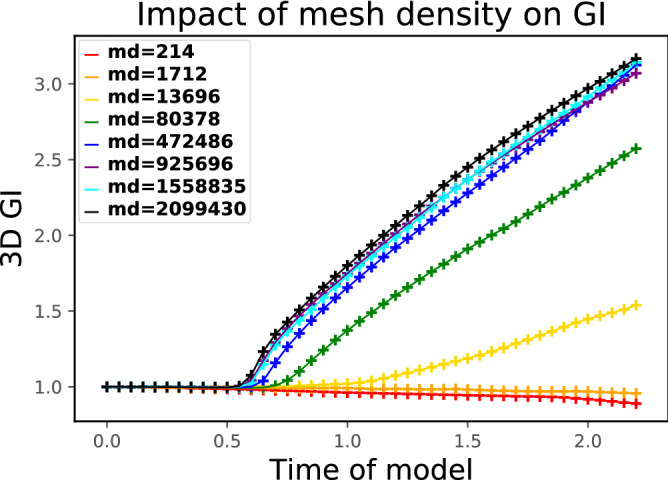
The comparison of 3D GI for simulated surfaces of different mesh densities (md).

The comparison of the 3D GI computed on surfaces of different mesh densities is shown in Fig. 12. The higher the mesh density is, the greater the 3D GI becomes, but for meshes with densities greater than the order of $10^{6}$ tetrahedra/cm$^{3}$, the difference in the 3D GI is very small (< 0.1) and the tendence is not evident. Considering both the mean curvatures and the 3D GI, the mesh with the density of $10^{6}$ tetrahedra/cm$^{3}$ can already achieve sufficient folding accuracy using the brain folding finite element model, thus this mesh density was used to study the impact of biophysical parameters onto folding patterns.
